# Effect of six weeks 1000 mg/day vitamin C supplementation and healthy training in elderly women on genes expression associated with the immune response - a randomized controlled trial

**DOI:** 10.1186/s12970-021-00416-6

**Published:** 2021-03-02

**Authors:** Małgorzata Żychowska, Agata Grzybkowska, Mariusz Zasada, Anna Piotrowska, Danuta Dworakowska, Olga Czerwińska-Ledwig, Wanda Pilch, Jędrzej Antosiewicz

**Affiliations:** 1 Department of Sport, Faculty of Physical Education, Kazimierz Wielki University in Bydgoszcz, Jana Karola Chodkiewicza 30, 85-064 Bydgoszcz, Poland; 2 Department of Biochemistry, Faculty of Physical Education, Gdansk University of Physical Education and Sport, Kazimierza Gorskiego 1, 80-336 Gdansk, Poland; 3 Institute for Basic Sciences, Faculty of Physiotherapy, University of Physical Education in Krakow, Jana Pawła II 78, 31-571 Krakow, Poland; 4 Department of Bioenergetics and Exercise Physiology, Faculty of Health Sciences with Institute of Maritime and Tropical Medicine, Medical University of Gdansk, Debinki 1, 80-211 Gdansk, Poland

**Keywords:** Supplementation, Aging, Genes involved in immune response

## Abstract

**Background:**

In this study, we investigated the effects of supplementation and exercise on the expression of genes associated with inflammation like *CCL2*, *CRP, IL1*, *IL6*, *IL10* mRNA in elderly women.

**Methods:**

Twenty four participants divided randomly into two groups were subjected to 6 weeks of the same health training program (three times per week). SUP group (supplemented, *n* = 12, mean age 72.8 ± 5.26 years and mean body mass 68.1 ± 8.3 kg) received 1000 mg of Vitamin C/day during the training period, while CON group (control, *n* = 12, mean age 72.4 ± 5.5 years and body mass 67.7 ± 7.5 kg) received placebo.

**Results:**

No significant changes in *IL-1*, *IL-6*, *IL-10* and *CRP* mRNA were observed within and between groups. However, there was a clear tendency of a decrease in *IL-6* (two-way ANOVA, significant between investigated time points) and an increase in *IL-10* mRNA noted in the supplemented group. A significant decrease in *CCL2* mRNA was observed only in the CON group (from 2^0.2 to 2^0.1, *p* = 0.01).

**Conclusions:**

It can be concluded, that 6 weeks of supplementation and exercise was too short to obtain significant changes in gene expression in leukocytes, but supplementation of 1000 mg vitamin C positively affected *IL-6* and *IL-10* expression – which are key changes in the adaptation to training. However, changes in body mass, *IL1* and *CCL2* were positive in CON group. It is possible that Vitamin C during 6 weeks of supplementation could have different effects on the expression of individual genes involved in the immune response.

**Trial registration:**

Retrospectively registered.

## Introduction

Despite many years of research on the impact of antioxidant supplementation on human health, until now there has been no clear opinion confirming or denying the need for supplementation in healthy subjects. It seems to be obvious that antioxidants should influence antioxidative status in the body. Therefore, supplementation should be effective in any case involving a possible increase in oxidative stress, for example in diseases (i.e. cardiovascular, cancer and others) [1, 2]. Increase in oxidative stress and inflammation also occurs during physical activity [3] and aging [4]. Thus, it seems to be an easy way to improve exercise tolerance as well as a preventive therapy against aging [5]. Unfortunately, in the literature the data associated with helpful actions of antioxidant supplementation are ambiguous. For example, Righ et al. [6] and Chou et al. [7] postulated that vitamin C supplementation attenuates both oxidative stress and inflammation in young males as well as in Taekwondo athletes. Some studies have shown that despite an increase in antioxidative capacity following vitamin C supplementation, pro/antioxidative status did not change [3]. No changes in the pro/antioxidative balance were associated with the possible pro-oxidative effect of vitamin C [3, 8]. A decrease in inflammation could disturb normal adaptations to training. According to Mankowski et al. [9], exercise-induced oxidative stress is an important trigger to induce training adaptations. The data from literature in which supplementation was investigated is ambiguous. There are many reasons for differences in the literature data, including different responses to supplementation depending on the used dose, type of exercise and age of participants [10–12].

The fast development of biomedical sciences has increased the average duration of human life, especially in developed countries. Aging is strongly associated with an increase in oxidative stress and inflammation [13]. According to Bektas et al. [14] in most aging individuals, a chronic low-grade pro-inflammatory state could be observed, and this factor can influence physical and psychological dysfunction. For this reason, in the current literature, there are two leading concepts: healthy aging and inflammation aging [15, 16]. Chronic inflammation could be a cause of inflammation aging and promote the development of many diseases such as neurodegenerative disease, cancer and others [17, 18]. Unfortunately, an imbalance between pro and antioxidative status accompanies both aging and physical activity. It seems that vitamin C supplementation could be especially helpful for older people subjected to physical effort due to a reduction in prolonged oxidative stress, and indirectly, inflammation.

Changes in oxidation status are important for cell survival. It is the main factor that could influence the expression of genes associated with cellular stress response, e.g., genes encoding heat shock proteins and interleukins [19, 20]. Cell survival under stressful conditions depends on a balance between activation of pro and anti-apoptotic pathways [21]. Unfortunately, in the literature, there are not many studies in which changes in genes expression caused by supplementation and exercise was investigated, especially in older participants. Recently, we investigated changes in expression of genes associated with iron metabolism, which is also easily induced by oxidative stress in elderly women (part of a broader project on vitamin C supplementation during health training). We concluded that vitamin C supplementation caused a decrease in ferritin mRNA [3]. Indirectly these findings indicate that vitamin C supplementation decreased intracellular free iron and consequently oxidative intracellular status. If this is true, the expression of genes associated with immune response, such as *CCL2*, *CRP*, *IL-1*, *IL-6* and *IL-10* should change. These genes encode proteins easily induced by oxidative stress and physical exercise and have great importance for immune response and adaptation to exercise. For example, monocyte chemoattractant protein 1, (CCL2 - chemokine (C-C motif) ligand 2) is capable of recruiting monocytes, memory T cells, and dendritic cells to induce a pro-inflammatory response and is produced by tissue injury [22, 23]. C-reactive protein (CRP - encoded by *CRP* gene) is a non-specific marker of ongoing inflammation, tissue damage and necrosis. IL-1 and IL-6 are pro-inflammatory proteins (IL-6 is inhibited by the expression of anti-inflammatory IL-10). According to Ziemann et al. [24] increase in anti-inflammatory proteins and a decrease in pro-inflammatory expression is crucial for adaptation to exercises.

Until now, most of the data in which inflammation, exercise and aging were investigated concerning plasma or muscle changes caused by vitamin supplementation, such as oxidative and antioxidative status, cortisol, CRP or IL-6 level only in plasma. Thus, the aim of our study was to investigate changes in oxidative stress, antioxidative capacity and the expression of chosen genes associated with inflammation, during 6 weeks of health training supported and unsupported by daily 1000 mg vitamin C supplementation in elderly women. We assumed, that this supplementation could cause a decrease in intracellular oxidation and a decrease in expression of proinflammatory cytokines mRNA. We also expected an increase in *IL-10* mRNA as a positive effect of vitamin C supplementation during training in older participants.

## Methods

### Ethics

All procedures were approved by the Bioethics Committee at the Regional Medical Chamber in Gdansk (KB-10/16). The study protocol was constructed according to the Declaration of Helsinki, and all participants gave their written consent, were fully informed prior to participation, informed about the possibility of withdrawal at any time for any reason and were given the opportunity to view their results.

### Subjects

From the group of 40 volunteers, 24 women meeting the inclusion criteria were selected. Only women at the age of 65 years and older, with BMI under 25, not taking any medications on a permanent basis, not smoking or having any other addictions. None of the women took any supplements for 3 months prior to the experiment. Only those qualified by a sports medicine doctor took part in the experiment. The women were subjected to tests typical for athletes, i.e. general medical interview, electrocardiogram, blood pressure and heart rate measurements, as well as basic laboratory determinations (morphology, urine, blood glucose, cholesterol). Participants were divided randomly into two groups: supplemented group (SUP) *n* = 12, mean age 72.8 ± 5.26 years, mean body mass 68.1 ± 8.3 kg, and control group (CON) *n* = 12, mean age 72.4 ± 5.5 years, body mass 67.7 ± 7.5 kg. All participants have never been professional athletes. The participants did not know who received vitamin C supplementation or cellulose (products in caps, look similar, double-blinded).

Body mass analysis was performed using the InBody 720 composition analysis (In body, Department Poland). The basic characteristics of the SUP and CON are summarized in the Results section (Table 1). To eliminate women with poor diets we also performed diet analysis and participants were asked not to change their nutritional habits during the study.

**Table 1:** Anthropometric characteristics of participants before and after 6 weeks of training supported (SUP group) or not supported (CON) Vitamin C supplementation

Parameter	Group	I	II	*p* I vs. II	ANOVA 2-way	dCohen I	dCohen II
VO_2_ max. [ml/kg/min]	SUP	21.4 ± 3.6	22.1 ± 3.4	0.2	row factor^*^	0.23	0.03
	CON	22.2 ± 3.5	22.00 ± 3.5	0.3			
*p1* (SU PI vs CON I) = 0.3; *p2*(SUP II vs CON II) = 0.4							
Body mass [kg]	SUP	68.1 ± 8.3	67.0 ± 7.9	0.30	row factor, time^*^	0.05	0.42
	CON	67.7 ± 7.5	63.8 ± 7.5^*#^	0.01			
*p1* (SUP I vs CON I) = 0.30; *p2* (SUP II vs CON II) = 0.02							
Muscle mass [kg]	SUP	22.4 ± 2.3	23.3 ± 2.6^#^	0.79	row factor^*^	0.25	0.11
	CON	23.0 ± 2.5	23.6 ± 2.7	0.33			
*p1* (SUP I vs CON I) = 0.01; *p2*(SUP II vs CON II) = 0.36							
Fat mass [kg]	SUP	26.6 ± 7.1	24.2 ± 7.2	0.09	row factor, time^*^	0.44	0.46
	CON	23.4 ± 7.5^#^	20.8 ± 7.6^*#^	0.02			
*p1* (SUP I vs CON I) = 0.04; *p2* (SUP II vs CON II) = 0.02							

^*^Significant differences (*p* ≤ 0.05) between I and II within the groups

^#^Significant differences (*p* ≤ 0.05) between groups

### Training

The training program was similar to the one used and described earlier [3]. Mainly the principles of health training were followed. Women participated in a 6-week multidisciplinary training program (three times per week for 60 min in duration) consisting of gyrokinesis, stabilization training, and Nordic walking at moderate intensity. Total duration of the training period overall was 1080 min. Heart rate was monitored during each training session (Polar H1). Participants were asked not to exceed a heart rate of 130/min.

### Supplementation

During the 6 weeks of training, the SUP group received 1000 mg of vitamin C (Max VitaC 1000, Colfarm, Poland) and CON group received cellulose in tablets (Colfarm, Poland). People conducting the experiment and participants did not know who was receiving supplementation and who was receiving placebo. The choice of vitamin C dose was chosen due to its easy availability and high frequency of consumption in the Polish population.

### Determination of VO_2_ max

The VO_2_ max was determined using the cycloergometer Ergoline Ergoselect 150p (Jaeger OxyconPro) and a gas analyzer (Jaeger OxyconPro) using the direct method. The measurement procedure was as follows: 2 min for registration of resting values followed by 5 min of a warm-up with 30 W load and 60 rpm cadence. Participants performed the test with the load gradually increasing by 10 W/min. The test was stopped if the participant was unable to continue with a given power and the required term of 60 rpm, or if symptoms occurred which indicated the need to end the trial. The results of VO_2_ max testing are presented in Table 1.

### Blood collection, analysis of vitamin C concentration, total oxidative status (TOS)/total oxidative capacity (TOC), total antioxidative status (TAS)/total antioxidative capacity (TAC) analysis and analysis of gene expression

Blood samples were collected twice: immediately before and 24 h after the training period. Venous blood (5 mL) was collected into BD Vacutainer tubes (Becton Dickinson, USA), in order to evaluate TOS/TOC and TAS/TAC. Plasma was separated by centrifugation (3000 g at 4 °C for 10 min). Collected plasma was stored immediately at − 80 °C for further analysis. We used photometric PerOx assay kit to evaluate TOS/TOC status (Immundiagnostik AG, Germany), and ImAnOx assay kit (Immundiagnostik AG, Germany) for TAS/TAC analysis. From the obtained results prooxidative/antioxidative balance was calculated.

### Vitamin C plasma concentration measurement

Vitamin C concentration was determined in plasma using the method of Robitaille and Hoffer [25] described in our earlier paper [3].

### Genetic evaluation

The protocol used for gene expression evaluation has been previously described in detail by Grzybkowska [26], Żychowska [3]. Briefly, 2 ml of venous blood was collected and to remove red blood cells, the Red Blood Cell Lysis Buffer (RBCL) (A&A Biotechnology, Gdynia, Poland) was added and the samples were then centrifuged. Obtained leukocytes were lysed using Fenozol (A&A Biotechnology, Gdynia, Poland) and stored at − 20 °C. The isolation of total RNA was performed based on Chomczynski and Sacchi method [27] using 200 μL of chloroform (POCH, Gliwice, Poland), centrifugation, and 500 μL of isopropanol (POCH, Gliwice, Poland). The obtained pellet was washed twice in 1 ml of 75% ethanol and spun at 7500 g at 4 °C. The dried RNA was then resuspended in the PCR-grade water, and after spectrophotometric evaluation of the purity and concentration of obtained material, the reverse transcription procedure was performed using 1000 ng of pure RNA, 0.2 μM oligo (dT) and the Transcriptor First Strand cDNA Synthesis Kit as per the manufacturer’s instructions (Roche, Warszawa, Poland). Immediately after this step, the samples were frozen and stored at − 20 °C without additional freeze-thaw cycles. For the quantitative real-time polymerase chain reaction (qRT-PCR) step, the 1:10 dilution of the cDNA has been used. This step was performed on AriaMx Real-Time PCR System (Agilent Technologies, Warszawa Poland) using FastStart Universal SYBR® Green Master (Rox) (Roche, Warszawa, Poland) according to the manufacturer’s protocol. Three replicates of 2 μL of diluted cDNA were used for qRT-PCR analysis. For each reaction, the melt curve analysis was performed to check for non-specific amplification.

The *TUBB* was used as a reference gene. All primers sequences were designed by authors in the Primer3 web tool and then in silico PCR tool in the USCS genome browser was used. All primers were delivered by Genomed, Warszawa, Poland. Primer sequences (5′-3′) used to perform this experiment were as follows:

For *TUBB* (tubulin beta class I, NM_001293213): Forward primer: CTAGAACCTGGGACCATGGA and Reverse primer: TGCAGGCAGTCACAGCTCT.

For *IL1* (NM_000575.5): Forward primer: AGT GCT GCT GAA GGA GAT GCC T and Reverse primer: CCT GCC AAG CAC ACC CAG TAG.

For *IL6* (NM_000600.5): Forward primer: AAT TCG GTA CAT CCT CGA CGG and Reverse primer: GAA TCC AGA TTG GAA GCA TCC.

For *IL10* (NM_000572.3); Forward primer: GAC ATC AAG GCG CAT GTG AAC Reverse primer: TCC ACG GCC TTG CTC TTG TTT.

For *CCL2* (NM_002982.4); Forward primer: CAG CCA GAT GCA ATC AAT GCC Reverse primer: CTTGGCCACAATGGTCTTGAA.

For *CRP (*NM_000567.3): Forward primer: TCG TTA ACG GTG CTT TGA GG and Reverse primer: TCT TGG TCT TGA CCA GCC TCT.

### Statistical analyses

First, the presence of a normal distribution was checked with the Shapiro-Wilk’s test for all results. Data obtained before and after 6 weeks of training period were compared within each group using a paired test. The differences between groups were analyzed using parametric or non-parametric (Wilcoxon) tests, as appropriate, as well as two-way ANOVA. The pro and antioxidative balance were calculated as the pro/anti ratio. Relative expression was calculated in Microsoft Excel 2015, using Schmittgen and Livak’s method [28]. Data were transformed into linear values, and subjected to the same methodology as the other parameters. To determine the significance of differences between the groups in gene expression, t-test and two-way ANOVA were used. Calculations and figures were generated using GraphPad Prism 6.0 software. *P*-values were considered statistically significant when ≤0.05.

## Results

### Changes in body composition and VO_2_ max

Despite similar total body mass in both groups, significant differences were observed in muscle mass and fat mass before the experiment (Table 1). Fat mass in CON group was significantly lower after the training period, with 6 weeks of training causing a significant decrease in total body mass and fat mass only in CON group. Therefore, significantly lower total body mass and fat was observed in CON group after 6 weeks of training without supplementation. No significant changes in VO_2_ max were noted in either group. However, only body mass after intervention showed a medium effect size (dCohen increase to 0.42).

**Fig. 1:**
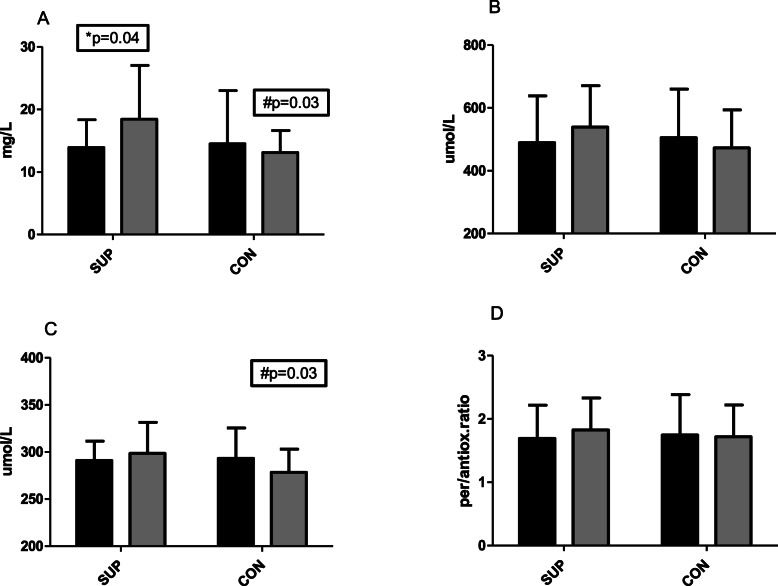
Changes in Vitamin C concentration (**a**), prooxidative status (**b**), antioxidative status (**c**) and pro/antioxidative ratio (**d**) before (dark bars) and after (gray bars) 6 weeks of training. SUP - participants trained and supplemented 1000 mg of Vitamin C. CON- participant only trained. *significant differences (*p* ≤ 0.05) between I and II within the groups; #significant differences (*p* ≤ 0.05) between groups

After 6 weeks of supplementation, vitamin C concentration increased in the SUP group from 13.9 ± 4.2 mg/L to 18.4 ± 8.6 mg/L, *p* = 0.04 (dCohen showed a strong effect size at the level 0.81). At the end of the experiment, there was a significant difference in plasma vitamin C concentration between groups (18.4 ± 8.58 mg/L in SUP and 13.1 ± 5.2 mg/L in CON, *p* = 0.03, Fig. 1a). These changes did not significantly influence antioxidative and prooxidative capacity however, there was a minor tendency to increase prooxidative capacity observed in the SUP group (from 489.4 ± 149.4 to 538.7 ± 132.1umol/L). This tendency was not observed in the CON group. Despite no significant changes in antioxidative capacity, the slight tendency to increase in SUP and decrease in CON caused a significantly lower antioxidative status in CON (278.4 ± 24.6umol/L in CON and 298.5 ± 32.9umol/L in SUP, *p* = 0.03). A consistently similar tendency in pro/antioxidative ratio was observed with a slight tendency to increase in the SUP group. Effect size (dCohen was strong for prooxidative and antioxidative status, 0.52 and 0.70, respectively.

**Fig. 2:**
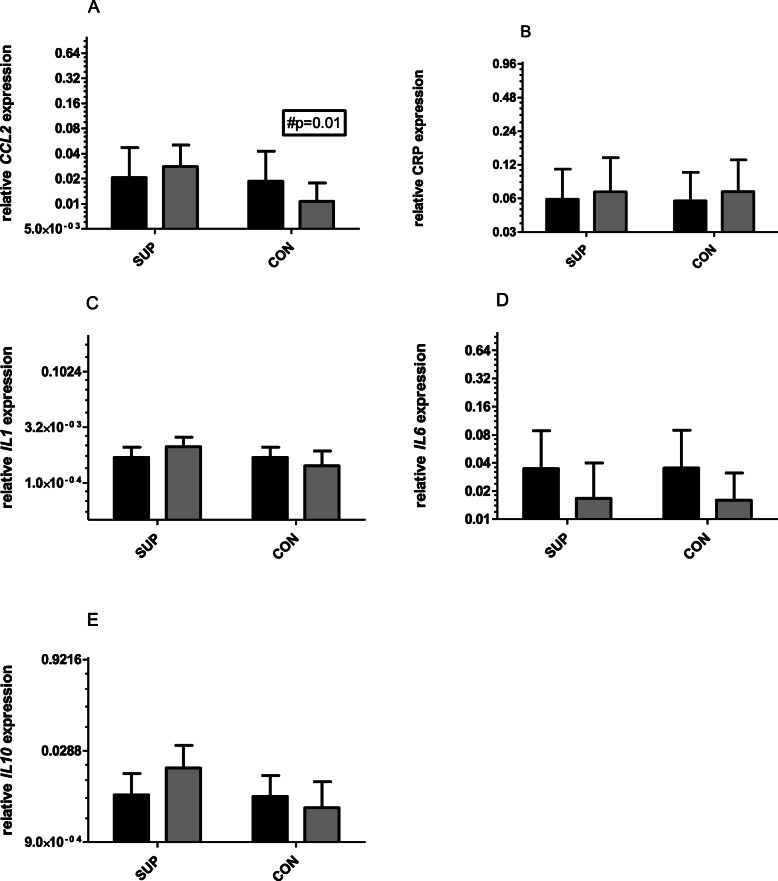
Changes in relative expression (2ˆ) of *CCL2* (**a**), *CRP* (**b**), *IL1* (**c**), *IL6* (**d**) and *IL10* (**e**), before (darky bars) and after 6 weeks (gray bars) training. All mRNA expressed as 2ˆ relative expression/TUBB. # significant differences between the group

There were no significant changes in gene expression for all tested genes observed after 6 weeks of training supported by 1000 mg vitamin C supplementation (Fig.2.). There was a tendency for a decrease in *IL-6* mRNA levels (from 2^0.035 to 2^0.016, two-way ANOVA, significant for time) and an increase in *IL-10* mRNA levels (from 2^0.005 to 2^0.015) was noted for SUP group. A similar tendency in decrease in *IL-6* mRNA occurred in CON (change from 2^0.035 to 2^0.015). In both groups, *CRP* and *IL-1* mRNA remained unchanged. There was a significant decrease in *CCL2* mRNA observed in the CON group (from 2^0.2 to 2^0.1, *p* = 0.01, two-way ANOVA, significant for time and subject). However, effect size (dCohen) was strong for three of tested genes: *IL1* mRNA (1.09), *CCL2* mRNA (1.33) and *Il10* mRNA (2.91) only at the end of the intervention.

## Discussion

In our research, we hypothesized, that vitamin C could increase antioxidant capacity and reduce oxidative stress and in this way lead to decrease in expression of genes induced by oxidative stress, such genes associated with inflammation. Unfortunately, our hypothesis has not been confirmed by research. In order to examine changes caused by vitamin C supplementation, we selected women similar in body mass. As a result, the dose of vitamin C per kg of body weight was similar within the supplemented group. Although we did not assume the differences in body composition at baseline and after 6 weeks of 1000 mg vitamin supplementation, our participants differed significantly in muscle mass and fat mass at baseline, despite no significant differences in total body mass and body mass index (BMI.) Moreover, a significant decrease in body mass and fat mass was observed in CON group after 6 weeks of training without supplementation (strong effect for total body mass). Thus, in the best case, vitamin C did not influence body mass and fat loss. Opposite data was reported by Johnston et al. [29]. The authors postulated that plasma vitamin C concentration inversely related to body mass and markers of obesity, however 500 mg of vitamin C supplementation did not influence the circulating concentration of adiponectin (necessary for lipolysis) in adult participants. The hindered reduction of fat mass observed in our participants receiving supplementation could be associated with the high dose of vitamin C supplementation (1000 mg/day). It is possible, that this supplementation affects IL-6 expression in adipose tissue, leading to a decrease in IL-6 expression. In the literature, there are some studies in which IL-6 is considered an important factor to induce fat loss by promoting white adipose tissue and browning lipolysis [30]. However, Wong et al. [31] postulated the negative relationship between intake of vitamin C and the risk of developing metabolic syndrome, associated with obesity.

As noted above, significant changes in body mass and fat mass, but not in muscle mass was reported in the CON group, subjected only to training. It is well documented that regular exercise increases the necessity to produce more ATP, influences metabolic rate [32, 33, and others], and finally is helpful in the reduction of body mass. However, in older subjects increase or no change in muscle mass is very important in maintaining the independence of older people [34, 35]. The effects of training are dependent on the type of exercise. Some authors postulated that during resistant training increase in muscle mass can only be achieved with the simultaneous good dietary approach diet [36]. In our experiment, there was no dietary intervention in CON group, thus the tendency for an increase in muscle mass in both groups should be regarded as a positive effect of applied training.

The concentration of vitamin C in the serum of our subject at baseline was average, and a significant increase was noted in SUP group after 6 weeks 1000 mg vitamin C supplementation (strong effect size). The final concentration was close to the upper limit of normal in the supplemented group. Unfortunately, an increase in vitamin C concentration was associated with a tendency to increase prooxidative and antioxidative status (strong effect size) and finally, we observed a tendency to increase pro/antioxidative balance. Generally, in both groups, six weeks of training supported or unsupported by vitamin C supplementation proved to be too short for any significant changes in pro or antioxidative status. Similar data after 6 weeks of supplementation during 12 weeks of training was observed in previously published data [3]. It seems, that 6 weeks of vitamin C supplementation in a dose of 1000 mg/day does not influence pro and antiinflammatory status, independently of body mass and BMI. Similar reports can be found in the literature. No changes in pro/antioxidative balance were reported by Bunpo and Antony [37], although their participants were healthy young men who received 250-500 mg vitamin C per day during 12 weeks of exercise. Moreover, the cited authors reported a decrease in antioxidative enzyme activity in erythrocytes. Assuming no influence of this supplementation on pro/antioxidative balance it is probable that vitamin C could show oxidative action, which is often indicated by other various authors [3, 38, 39]. It is possible, that vitamin C could promote the generation of reactive oxygen species i.e., OH, O_2_^−^, H_2_O_2_ or ferryl ion [3, 8].

In terms of the obtained results, changes in genes associated with an immune response may not be consistent with our assumptions. Our hypothesis, that vitamin C supplementation in a dose of 1000 mg/day will cause a decrease in proinflammatory and an increase in anti-inflammatory gene expression was not confirmed. First of all, no significant changes in gene expression of all studied genes were noted in the SUP group. Only in the CON group, *CCL2* mRNA significantly decreased after the training period. This gene is not often investigated underexercising conditions, despite its important functions in inflammation. Micielska et al. [40] reported a tendency for a decrease in *CCL2* expression in leukocytes after high-intensity circuit training in adult women. Moreover, cited authors connect this change with counteracting cognitive decline. Thus, it seems that different types of exercise affect *CCL2* expression in leukocytes, however, opposite changes in muscle cells were noted [41]. Overexpression of *IL-6* and *CCL2* could be a reason for the development of acute inflammation and inflammatory pain [42]. The most common changes in the *CCL2* expression in humans are indicated in peripheral blood mononuclear cells (PBMC) or leukocytes. According to Strömberg et al. [43] its expression in muscle cells was marginal. *CCL2* mRNA and CCL2 protein are responsible for macrophage induction. It is well documented, that *CCL2* released by astrocytes has an impact on cognitive dysfunction and brain inflammation [44, 45]. It means that a decrease in expression may have a positive impact on cognitive function and decrease brain inflammation. Thus, obtained results showed positive changes affected by training in those not supported by vitamin C supplementation.

In the literature, there is data regarding the influence of vitamin C supplementation mainly on plasma CRP. Biniaz et al. [46] reported that vitamin C supplementation is associated with a decrease in CRP in patients undergoing hemodialysis. Ellulu et al. [47] reported that vitamin C (500 mg twice daily) has potential effects in alleviating inflammatory status by reducing high-sensitivity C-reactive protein **(**hs-CRP) and IL-6in hypertensive and/or diabetic obese patients [48]. Unfortunately, their research was conducted on sick people, for which vitamin C supplementation may have different effects than in healthy people when subjected to exercise. Results of ultramarathon runners showed that vitamin C caused an increase in CRP by reducing the secretion of cortisol [49]. Our results (no changes in CRP mRNA in both groups) are consistent with the data published by Righi et al. [6]. The authors observed no changes in CRP following single both of acute exercise.

It is well documented that changes in IL-1 caused by exercise are less than another in cytokines, e.g. IL-6. The available data shows that the increase in IL-1 is dependent on training load. Significant increase in this cytokine was observed after ultramarathon, but only in people supplemented by 1500 mg of vitamin C (at a dose of 500 mg of vitamin C there was no such effect). This increase was small in comparison to IL-6. No changes in *IL-1* mRNA were observed in our study which confirms lower induction of this cytokine gene expression by physical exercise and no effect of 6 weeks of 1000 mg/day vitamin C supplementation.

In both groups, the same tendency to decrease in *IL-6* mRNA was observed. Thus, this effect is mainly associated with training. Our results are compatible with data from other literature studying the influence of aerobic exercise on the modulation of the cytokine profile. Although available data is mainly associated with changes in inflammatory (IL-6 or IL-1) protein measurement in plasma or serum. According to El-Kader and All Jiffri [50], a significant decrease in IL-6 and an increase in IL-10 was observed following 6 months of aerobic exercise in elderly subjects. Thus, 6 weeks of training may be too little to obtain significant differences, especially that we did not observe a tendency to increase in *IL10* mRNA in the control group. An increase in IL10 after training period was observed in several studies [24, 50, 51]. Moreover, Canali et al. [52] suggested a small effect of vitamin C supplementation on gene expression, especially those associated with immune response. In the cited research the authors investigated changes in gene expression in five volunteers supplemented high dose of vitamin C (1 g/day) over 5 days. They suggested that vitamin C plays important role in the modulation of *IL-10* mRNA during an inflammatory stimulus. We agree with these authors that in healthy, well-nourished participants, supplementation of vitamin C is “buffered” within a homeostatic physiological equilibrium. However, a tendency to increase IL-10 with a simultaneous decrease in IL-6 is important in terms of adaptation to training [24]. This was clear in the supplemented group, however, 6 weeks of supplementation did not significantly influence this change.

## Conclusion

Generally, six weeks of training with moderate intensity was insufficient to obtained significant increase in VO_2_ max. and it seems to be important result for planning the training of the elderly people. It seems that 1000 mg/day vitamin C supplementation inhibited changes in body composition in elderly women. Applied 6 weeks of health training did not influence oxidative/antioxidative balance however the marked prooxidative effect was noticeable. Moreover, the tendency to decrease *IL-6* and increase *IL-10* mRNA in the supplemented group could indirectly indicate that oxidative stress within cells was lowered. However, most strong effect size was observed for *IL1* and *CCL2* mRNA, but this observations require further research. As there was no significant changes in pro/antioxidative balance with simultaneous change in *IL-6* and *IL-10* mRNA this change could be considered a positive effect of 1000 mg vitamin C supplementation. However, these effects were not spectacular, and it is not known whether a longer period of supplementation would have had an effect on oxidative/prooxidative balance in plasma.

### Study limitations

Our study has some limitations. Despite randomization, differences in body composition made it difficult to interpret the results. Also, only one dose (1000 mg daily) of vitamin C was used, and its effect can be expected to be dose-dependent. Moreover, the results cannot be directly extrapolated to men and women of other ages.

## Data Availability

The datasets used and/or analysed during the current study are available from the first author: Małgorzata Żychowska (malgorzata.zychowska@ukw.edu.pl) on reasonable request.

## Abbreviations

*CCL2*: Gene encoding C-C Motif Chemokine Ligand
*CRP*: Gene encoding C-reactive protein
*IL1*: Gene encoding interleukin 1
*IL6*: Gene encoding interleukin 6
*IL10*: Gene encoding interleukin 10
CON: Control group
SUP: Supplemented group
TOS/TOC: Total oxidative status/total oxidative capacity
TAS/TAC: Total anti-oxidative status/total antioxidative capacity
*TUBB*: Gene encoding tubulin B
RNA: Ribonucleic acid
VO2 max: Maximal oxygen consumption
